# [Retracted] Knockdown of lncRNA NUTM2A-AS1 inhibits lung adenocarcinoma cell viability by regulating the miR-590-5p/METTL3 axis

**DOI:** 10.3892/ol.2025.15424

**Published:** 2025-12-08

**Authors:** Jie Wang, Jingyun Zha, Xiaolin Wang

Oncol Lett 22: 798, 2021; DOI: 10.3892/ol.2021.13059

Following the publication of this paper, it was drawn to the Editor's attention by a concerned reader that flow cytometric (FCM) assay data featured in Figs. 4D and 7D were strikingly similar to FCM data that have ultimately have been published in a number of other papers in different journals that were written by different authors at different research institutes.

Owing to the fact that the contentious data in the above article have been shown to be strikingly similar to data that have appeared elsewhere in other papers in the scientific literature, the Editor of *Oncology Letters* has decided that this paper should be retracted from the Journal. The authors were asked for an explanation to account for these concerns, but the Editorial Office did not receive a reply. The Editor apologizes to the readership for any inconvenience caused.

